# Fabrication, Mechanical Properties and In-Vitro Behavior of Akermanite Bioceramic

**DOI:** 10.3390/ma13214887

**Published:** 2020-10-30

**Authors:** Fariborz Tavangarian, Caleb A. Zolko, Sorour Sadeghzade, Marwan Fayed, Keivan Davami

**Affiliations:** 1 Mechanical Engineering Program, School of Science, Engineering and Technology, Pennsylvania State University, Harrisburg, Middletown, PA 17057, USA; caz5067@psu.edu (C.A.Z.); sxs2640@psu.edu (S.S.); 2 Department of Mechanical and Nuclear Engineering, Pennsylvania State University, University Park, PA 16802, USA; mqf5462@psu.edu; 3 Department of Mechanical Engineering, University of Alabama, Tuscaloosa, AL 35487, USA; kdavami@eng.ua.edu

**Keywords:** akermanite, ceramics, mechanical properties, nanocrystals, bioactivity

## Abstract

Pure nanocrystalline akermanite (Ca_2_MgSi_2_O_7_) powder was synthesized by mechanical activation with subsequent annealing of talc, calcium carbonate, and silicate powders as the initial materials. Powder samples were characterized by X-ray diffractometry (XRD), scanning electron microscopy (SEM), thermogravimetric analysis (TGA), and transmission electron microscopy (TEM) techniques. The results showed that pure nanocrystalline akermanite with a crystalline size of 35 nm was synthesized after ball milling the initial powders for 20 h with subsequent annealing at 900 °C for 1 h. Mechanical properties of bulk akermanite samples were studied as well. The results showed that the produced akermanite tablets sintered at 1200 °C for 5 h had a Young’s modulus of 3800 MPa, an ultimate compressive strength of 24.7 MPa, and a density of 2.489 g/cm^3^. The in-vitro behavior of the produced akermanite was evaluated by soaking the samples in an SBF solution. The results showed that the produced akermanite had the apatite formation ability on its surface and can be a good candidate for bone tissue engineering applications.

## 1. Introduction

In the medical field, there is a need for materials that can be used to bond with living bone to help repair bone defects within the human body. Akermanite (Ca_2_MgSi_2_O_7_) is an osteoinductive bioceramic that can promote bone repair [1,2,3]. Akermanite has shown favorable mechanical strength, excellent bioactivity, and degradation rates [4,5]. The bioactivity and degradation rate help promote ossification within bone defects [1]. Ca^2+,^ Mg^2+^, and Si^4+^ ions available in akermanite structure can enhance osteoblastic proliferation rates, increase collagen production, and stimulate bone growth [2,3,4,5,6]. These ions have a stimulatory effect on osteoblast growth [7,8]. Ca is one of the primary elements within the bone matrix [9]. Ca ions have been shown to induce osteoblast proliferations [10]. Xynos et al. [4] determined that Ca ions promoted osteoblast proliferation and chemotaxis through binding to G-protein coupled extracellular calcium sensing receptors. Si is involved in the early mineralization process of ossification [4]. The release of Si ions helps stimulate both osteogenesis and angiogenesis. Si ions encourage nodule formation and enhance mineralization, and this mineralization is done without the presence of dexamethasone and β-glycerophosphate [11,12]. Mg plays an important role in determining the dissolution rate of the ceramic [5,13]. Mg ions are also required for regulating the ossification process [3]. Webster et al. [14] showed that Mg ions enhanced osteoblast adhesion with hydroxyapatite. A deficiency in Mg within the body can slow down the bone formation and potentially lead to osteoporosis [15,16].

Akermanite has been prepared by various techniques. To produce akermanite powder, conventional methods such as combustion method [17] and sol-gel method [8,9] have been used. Bhatkar et al. [17] synthesized akermanite in a time efficient manner using combustion method. However, the final product was contaminated with Eu^2+^ due to the nature of the combustion method. Also, sol-gel method has been used extensively to produce akermanite powder; however, the expensive initial materials and the long processing time are some disadvantages of this techniques [8,9]. Sintering method has been used to produce bulk akermanite samples [18]. To produce ceramics with conventional sintering technique, the initial powders are pressed in a mold and then subjected to heat treatment cycles at high temperatures. Ventura et al. [19] produced akermanite bulk sample by sintering method at low temperatures (750 and 800 °C). However, they observed that the produced akermanite powders had some impurities.

In our previous study [19], we synthesized akermanite powder from magnesium carbonate, calcium carbonate, and silica. However due to the nature of the initial materials, pure akermanite could not be produced up to 1200 °C. In this paper, for the first time, akermanite was synthesized using talc as one of the starting materials. Talc is the softest material known [20]. As such, a ball milling method benefits from using talc because it is extremely soft and easily crushed. This allows the talc to be crushed with less wear to the balls and container. Talc as a base material allowed for the formation of nanocrystalline akermanite powders after the annealing procedure at lower temperatures. It was expected that using ball milling method as well as talc, as one of the initial materials, accelerate the rate of reactions to form a nanostructured akermanite compared to traditional synthesis methods. Furthermore, the mechanical properties and in-vitro behavior of the produced akermanite were investigated to evaluate the potential of this ceramic to be used in bone tissue engineering applications.

## 2. Materials and Methods

Talc, (Mg_3_Si_4_O_10_(OH)_2_) (99% purity, Sigma-Aldrich, lot# SZBB1580V), Calcium carbonate (CaCO_3_) (99% purity, Acros Organics, lot# A0369056), and silica (SiO_2_) (99% purity, Sigma-Aldrich, lot# SLBS7820) powders were used as the initial materials. Talc, Calcium carbonate, and silica were mixed with a molar ratio of 1:6:2. This provides the proper ratio of Ca, Mg, and Si for the final product. The mixed powders were ball milled by a planetary ball mill (Tencan, model XQM-1-A, Changsha, Hunan, China) with a rotational speed of the main disc equal to 500 rpm. Zirconia containers (with the height and diameter of 70 × 75 mm, respectively) with 5 large, 10 medium, and 10 small balls (10 mm, 5 mm, 2 mm diameters, respectively) were used in conjunction with the planetary ball mill. The ball to powder weight ratio was 10:1. The initial powders were ball milled in an air atmosphere for 1, 3, 6, 10, 15, and 20 h. The ball milled powders were subsequently heat treated for 1 h at various temperatures with the heating/cooling rate of 10 °C/min.

To evaluate the mechanical properties of akermanite ceramic, powders were pressed into tablets with the height and diameter of 1 cm each. These tablets were prepared from ball milled powders for 6, 10, and 20 h. Tablets were pressed with a pressure of 100 MPa and subsequently annealed at 1200 °C for 5 h.

A PANalytical Empyrean diffractometer with Cu Kα radiation (λ = 0.154056 nm) was used for X-ray diffractometry (XRD) to evaluate the initial powders, ball milled samples, and heat-treated samples. A range of 2θ, 20–80° with a step size of 0.026° and a time step of 30.6 s was used. XRD patterns were analyzed with PANalytical X’pert highscore. The crystallite size of akermanite powders was estimated using the Williamson–Hall method as follows [21]:

(1) βcosθ= Kλ/D+ εsinθ

where β is the full width at half maximum height of the peak, θ is the Bragg diffraction angle, K is the Scherrer constant (0.91), λ is the wavelength of the radiation used in the XRD test, D is the crystallite size, and ε is the average internal strain.

Scanning electron microscopy (SEM) evaluation was performed on heat treated samples using a Helios Nanolab 660 (ThermoFisher Scientific, Hillsboro, Oregon, USA), with acceleration voltages of 3–20 kV. Thermogravimetric analysis (TGA)/mass spectrometry (MS), using the Discovery Series TGA5500 machine (TA Instruments, New Castle, DE, USA), was used to determine the temperatures of reactions along with the magnitude of weight loss associated with these reactions and the materials released from the system. The samples were evaluated from room temperature to 1000 °C utilizing a heating rate of 10 °C/min. Transmission electron microscopy (TEM) technique was used to evaluate the akermanite samples synthesized after 20 h mechanical activation with subsequent annealing at 900 °C for 1 h using an FEI Talos TEM.

Mechanical properties of akermanite tablets were studied with a compression test. Tablets were tested using an MTS Insight (Electromechanical – 30kN Standard Length). Furthermore, density and porosity of the samples were studied using DahoMeter DE-120M densimeter. The bulk density and porosity were determined according to the following equations [22]:

The density and porosity of samples were determined according to the following equations.

(2) $$\rho_{m}=\frac{w_{1}}{w_{1}-w_{2}}$$

(3) $$\mathrm{Total}\;\mathrm{porosity}=\left(1-\frac{\rho_{m}}{\rho_{\mathrm{th}}}\right)\times 100$$

where w_1_ is the dry weight of the tablets, w_2_ is the suspended wet weight of the tablet in water, $\rho_{m}$ is the measured density and $\rho_{\mathrm{th}}\;$is the theoreticaldensity.

The apatite formation ability of akermanite was evaluated in simulated body fluid (SBF). The samples were immersed in 15 cc SBF for 1, 7, 14, 21, and 28 days. Samples were kept at Benn Mary bath at 37 °C. The wet samples were dried at 40 °C for 5 h. The pH changes of the SBF solution as a function of time were measured up to 28 days. SEM, EDS, and XRD were utilized to evaluate the apatite formation ability on the surface of akermanite after soaking the samples for various time points in SBF.

All data were examined as average values ± standard deviation for n = 3. Data was analyzed for statistically significant differences with one-way ANOVA.

## 3. Results and Discussions

### 3.1. Thermogravimetry/Mass Spectrometry Analyses

**Figure 1:**
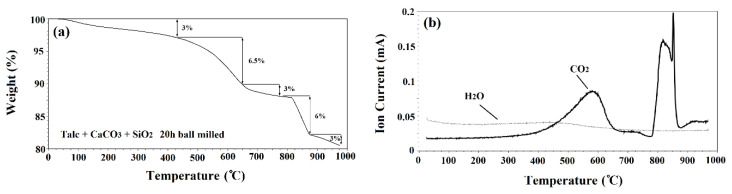
(**a**) TG and (**b**) mass spectrometry of powders after 20 h ball milling time.

Thermogravimetric analysis (TGA) and mass spectrometry (MS) were performed on 20 h ball milled powders. The results of TGA and MS are shown in Figure 1a,b, respectively. The TGA results showed two distinct steps of weight loss and the MS analysis revealed two main peaks. The initial peak related to CO_2_, began at 425 °C and diminished at 650 °C which corresponded with the first main weight loss step (6.5%). A sharper peak began at 775 °C and ended at 875 °C. This steeper peak corresponds with the second main weight loss step (6%) and was due to the release of CO_2_ from the system (see Figure 1b). There was also a steady release of H_2_O shown from 25 to 975 °C.

Of the initial powders, only talc and CaCO_3_ decomposed. Talc released structural H_2_O during decomposition [23]. Theoretically, 4.7% weight loss can be obtained from this decomposition. However, it was found that talc powder had a weight loss of 15% when heat treated up to 975 °C [23]. This increased weight loss was due to water absorbed by the powder from the atmosphere. CaCO_3_ releases CO_2_ during the decomposition with a theoretical weight loss of 44%. Galan et al. [24] found that crystalline CaCO_3_ with a purity above 99% decomposed with a weight loss of 43%. It has also been shown that both talc and CaCO_3_ can release absorbed H_2_O as well [25]. This could explain the consistent release of H_2_O throughout the TGA.

The TGA curve showed a total weight loss of 19.5% after annealing up to 1000 °C. However, the total theoretical weight loss of the powder was higher, equal to 25.6%. The difference between the theoretical weight loss and the experimental one could be ascribed to the release of volatile materials during the ball milling process. To test this, powder samples were weighed before and after 20 h of ball milling. The results showed 5% weight loss during the ball milling process. It was observed that after ball milling the powders for 20 h, gas came out of the jars with pressure when opening the lids of the ball mill containers which could be the cause of difference between the theoretical and experimental values. Similar observations regarding the difference between the experimental and theoretical values have been reported by other scientists [24]. Wieczorek-Ciurowa et al. [26] studied the effect of mechanical activation on TGA. They found that mechanical activation can affect the location, slope, number of steps and total weight loss in TGA curves. This is the cause of the step of weight loss at 500 °C to 650 °C and could also be another reason for the final weight loss being less than the theoretical value.

### 3.2. X-ray Diffraction Analysis

**Figure 2:**
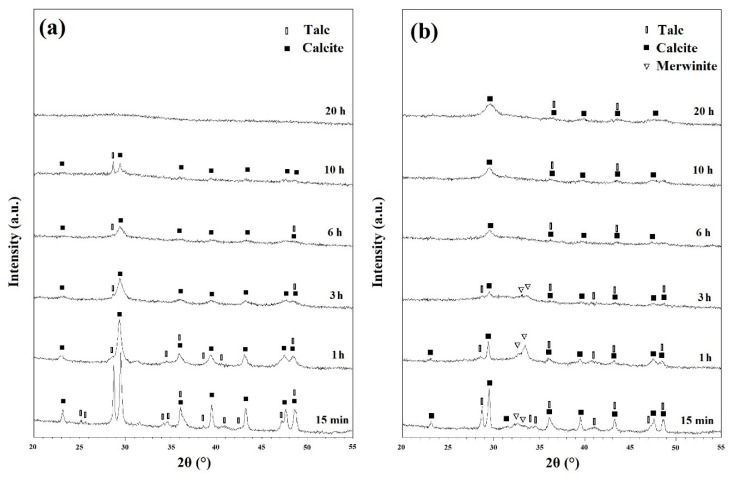
XRD patterns of ball milled powders (**a**) before and (**b**) after subsequent annealing at 700 °C for 1 h.

X-ray diffraction (XRD) analysis was performed to characterize the ball milled and heat-treated powders. Figure 2a depicts the XRD patterns of the ball milled powders before any heat treatment cycles. The powder ball milled for 15 min showed a distinct structure with sharp peaks. Talc (XRD JCPDS data file No. 00-013-0558) and CaCO_3_ (Calcite) (XRD JCPDS data file No. 00-005-0586) were both characterized distinctly. However, as can be seen from Figure S1, silica did not show any peaks on the XRD patterns. Further ball milling began to shorten and widen the peaks as a result of induced stresses, reduced grain size, and developing an amorphous state [27]. Once the powders have been ball milled for 20 h, they have become amorphous without any distinct peaks. After heat treatment the ball milled powders at 500 and 600 °C for 1h, there were only minor changes in the peaks which can be seen in Figures S2 and S3, respectively.

Figure 2b depicts the XRD patterns of milled powders which were annealed at 700 °C for 1 h. Those samples ball milled for 15 min, 1 h and 3 h, showed Ca_3_Mg(SiO_4_)_2_ (merwinite) phase (XRD JCPDS data file No. 00-025-0161) on their XRD patterns. However, with increasing the ball milling time, no new phase was observed which demonstrated that 700 °C is not enough temperature to advance the reactions kinetically and to develop new phases. Based on the results of the TGA/MS curves (Figure 1), talc and CaCO_3_ partially decomposed in samples heat treated at 700 °C which resulted in the formation of merwinite phase.

**Figure 3:**
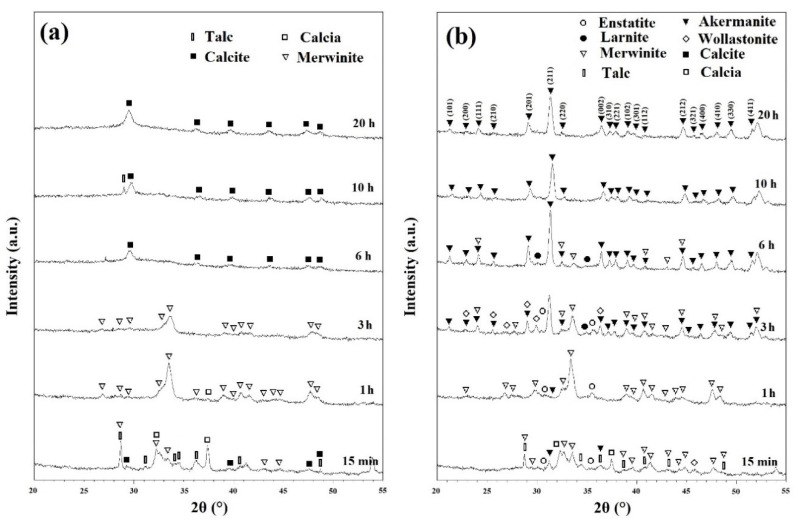
XRD patterns of powders after various ball mill times with subsequent annealing at (**a**) 800 °C and (**b**) 900 °C for 1 h.

Figure 3a shows the XRD patterns of ball milled powders for various times after annealing at 800 °C for 1 h. As discussed in Figure 1b, CaCO_3_ decomposed in two main steps: Step one from 425 to 650 °C and step two from 775 to 875 °C. In these samples, the remaining CaCO_3_ compound decomposed into CaO (calcia) (XRD JCPDS data file No. 00-037-1497) according to the following reaction [25]:

(4) $${\mathrm{CaCO}}_{3}\;\to \;\mathrm{CaO}\;+{\;\mathrm{CO}}_{2}.$$

CaCO_3_ has been reported to decompose at 650 °C [25]. However, it is clear that the rate of this decomposition is too slow at this temperature. This is in a good agreement with the results obtained from Figure 1. At this step, the intensity of characteristic peaks of merwinite phase began to increase within the powder (Figure 3a).

Figure 3b shows the XRD patterns of powders after various ball milling times with subsequent annealing at 900 °C for 1 h. Talc began to decompose into MgSiO_3_ (enstatite) phase (XRD JCPDS data file No. 00-003-0520), SiO_2_, and water at this temperature even without significant milling time. The decomposition of talc can be expressed as follows [28]:

(5) $${\mathrm{Mg}}_{3}{\mathrm{Si}}_{4}O_{10}{\left(\mathrm{OH}\right)}_{2}\to \;3{\mathrm{MgSiO}}_{3}+{\mathrm{SiO}}_{2}+{\;H}_{2}O.$$

Minor enstatite peaks were observed on the patterns of ball milled samples for 15 min, 1, and 3 h. Further milling removed these peaks due to the consumption of these phases to produce new intermediate compounds. Other phases that were detected in the XRD patterns were CaSiO_3_ (Wollastonite) (XRD JCPDS data file No. 00-010-0489) and Ca_2_SiO_4_ (larnite) (XRD JCPDS data file No. 00-002-0866).

The synthesis of Ca_2_MgSi_2_O_7_ (akermanite) (XRD JCPDS data file No. 01-077-1149) began in mechanically activated powders after annealing at 900 °C for 1 h. Akermanite became the dominant phase in 3 h ball milled powders, and pure akermanite was achieved after 10 and 20 h of mechanical activation at this temperature. Further annealing temperature to 1000 °C for 1 h (as shown in Figure S4), did not show any significant changes in the XRD patterns.

Mechanical activation has several notable effects on the XRD patterns. Filio et al. [27] showed that mechanical activation enhances the rate of reactions. The decrease in particle size, and the increase in internal stresses and grain boundaries caused by ball milling have a positive effect on the rate of reactions [29]. This is due to the nature of diffusion-based reactions where lower distances of diffusion paths results in quicker reactions and more homogenous products as observed in the XRD patterns of this study. XRD patterns shown in Figure 2 depict the visible effect of the decrease of crystallinity caused by ball milling. The shortening and widening of the peaks are directly caused by the decrease in crystallinity and increase in homogeneity [27,30]. Changes in reaction rates caused by mechanical activation can be seen distinctly in Figure 3b. Without significant ball milling, only minor amounts of akermanite was obtained within 1 h of annealing. With 3 h of ball milling, akermanite became the dominant phase; and further increase of ball milling changed the kinetics sufficiently to synthesize pure akermanite. The Williamson–Hall method was used to calculate the crystallite size of akermanite powder [21]. The calculated crystallite size was 34 nm for powders synthesized after 20 h ball milling with subsequent annealing at 900 °C for 1 h.

### 3.3. SEM and TEM Evaluation

**Figure 4:**
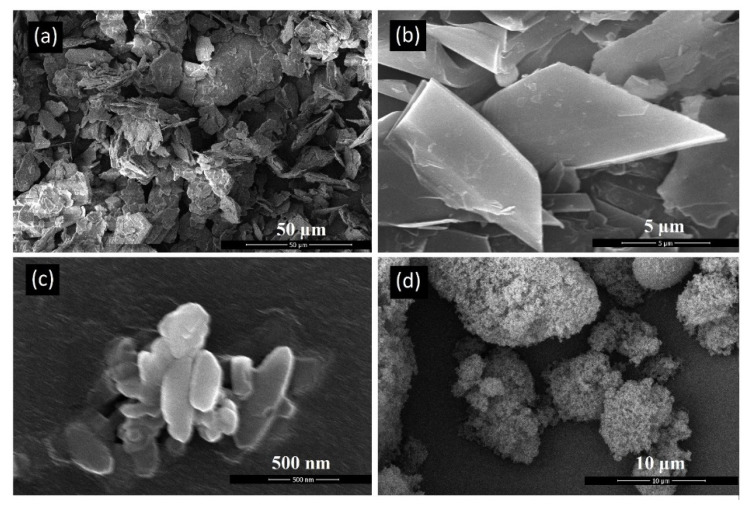
SEM micrographs of the initial powders (**a**,**b**) talc, (**c**) calcium carbonate, and (**d**) silica.

Scanning Electron Microscopy (SEM) was performed to characterize the akermanite powder as well as the initial materials. Figure 4a–d depicts an SEM micrograph of base materials including talc (Figure 4a,b), calcium carbonate (Figure 4c), and silicate (Figure 4d). Talc powders composed of thin sheets with a width of approximately 25 µm. Calcium carbonate particles were agglomerated rods approximately 1 µm in length. Silica powders was round in shape and highly porous with an average diameter of 10 µm.

**Figure 5:**
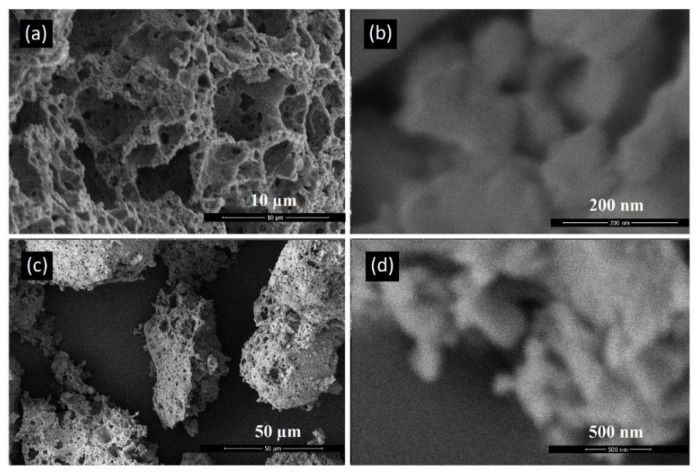
SEM micrographs of akermanite powders produced after ball milling the initial materials for (**a**,**b**) 10 h and (**c**,**d**) 20 h with subsequent annealing at 900 °C for 1 h.

Figure 5 depicts akermanite produced in this study. Akermanite powder particles were roughly round in shape and had a microporous structure. These structures were made up of particles that had been sintered together. Sintered particles, from akermanite powders synthesized from 10 h of milling, had a size ranging from approximately 25 to 300 nm. Powder particles synthesized from 20 h of milling were similar in size ranging from approximately 50 to 300 nm. Particles were made up of nanocrystals with the size of 34 nm for 20 h ball milled powders. This result means that particles are made up of numerous agglomerated nanocrystals.

**Figure 6:**
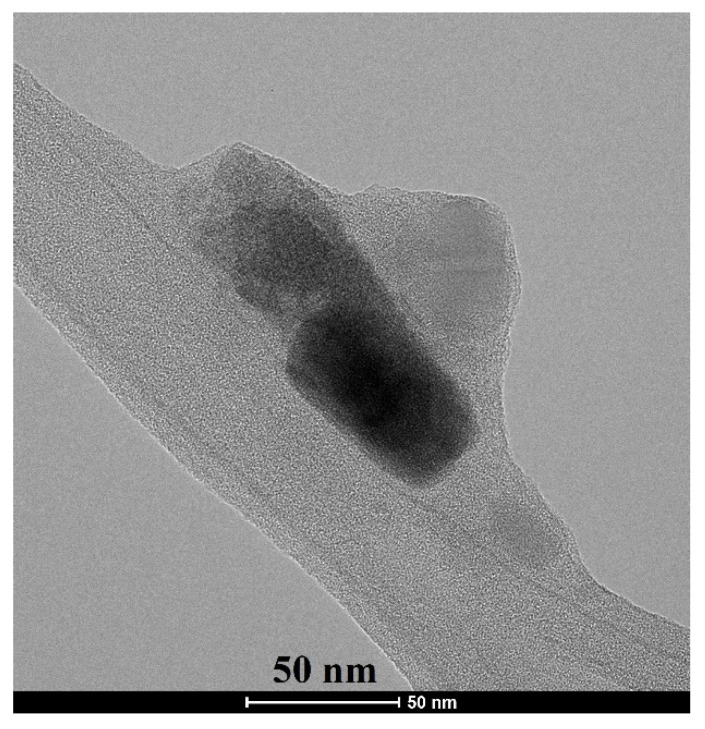
TEM micrograph of akermanite powder produced after 20 h ball milling of the initial materials with subsequent annealing at 900 °C for 1 h.

The crystallite size and morphology of akermanite powder obtained from the ball milling of initial materials for 20 h with subsequently annealed at 900 °C for 1 h can be seen in Figure 6. Crystallites were round and had been agglomerated. The size of crystallites was between 20 and 80 nm, which was in a good agreement with the results obtained from the Williamson–Hall method.

### 3.4. Mechanical Properties Evaluation

**Figure 7:**
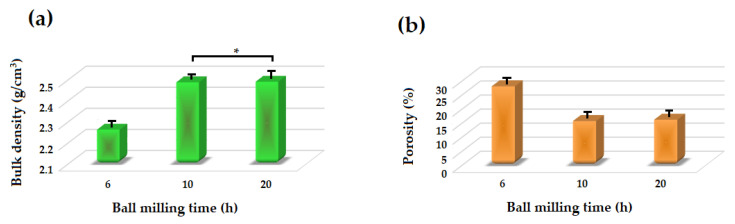
(**a**) Bulk density and (**b**) apparent porosity of different samples (* not significantly different).

Figure 7 depicts the density and porosity of the akermanite tablets. With increasing the ball milling time, the density of the tablets increased while their porosity decreased due to the release of more gases during the ball milling process and subsequently less volatile materials left the system during the sintering procedure. Also, with increasing the ball milling time more uniform powders were formed which caused the formation of pure akermanite bioceramic. During the sintering process, the release of vacant sites from the tablets caused an approximately 15% shrinkage in the tablets. The density of the samples was highest at 2.487 and 2.489 g/cm^3^ for the ball milled samples for 10 and 20 h, respectively.

**Figure 8:**
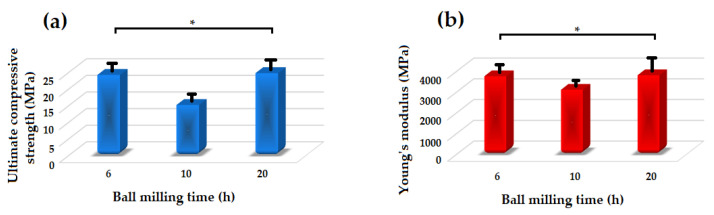
Mechanical properties of the akermanite tablets. (* not significantly different).

Figure 8 shows the Young’s modulus and ultimate compressive strength of the akermanite tablets. Those samples which are not significantly different according to the results of the statistical analyses are illustrated with asterisk. As seen, with increasing the ball milling time from 6 to 10 h the compressive strength and Young’s modulus decreased and then increased in 20 h ball milled sample. The samples ball milled for 6 h and 20 h had the highest Young’s modulus of approximately 3750 and 3800 MPa, respectively. Additionally, these specimens had the highest compressive strength of 24.15 and 24.7 MPa as well. As can be seen in Figure 3b, merwinite phase was observed in the ball milled samples for 6 h. The presence of a secondary phase can act as a reinforcement and improve the mechanical properties of the structure. With increasing the ball milling time to 10 h, only akermanite remains in the structure and the merwinite vanished which consequently decreased the compressive strength of the samples. With increasing the ball milling time, the effect of mechanical activation became dominant on the mechanical properties of the samples which caused the formation of smaller crystallite size and an increase in the compressive strength and Young’s modulus of the specimens.

### 3.5. Bioactivity Evaluation

**Figure 9:**
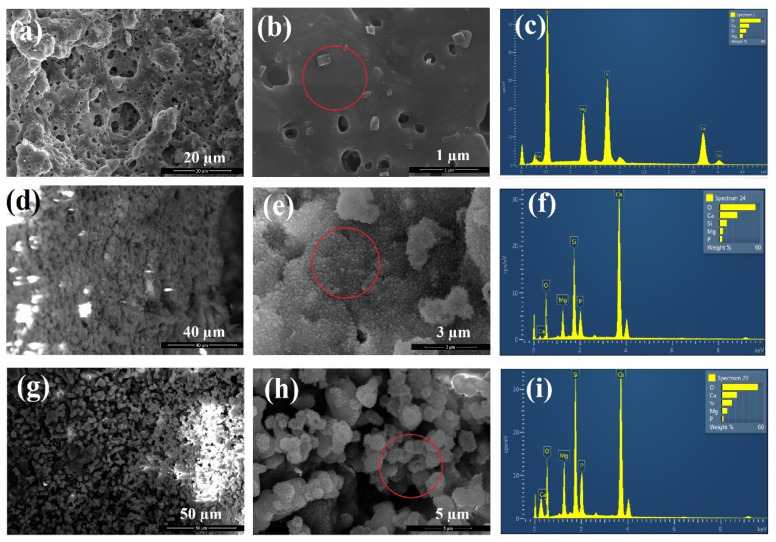
SEM images and EDS spectra of akermanite obtained from 20 h ball milled samples with subsequent annealing at 900 °C for 1 h (**a**–**c**) before, (**d**–**f**) after 14 days and (**g**–**i**) after 28 days soaking in SBF solution.

Figure 9 shows the SEM images and EDS spectra of akermanite powders obtained after 20 h ball milling of the initial materials with subsequent annealing at 900 °C for 1 h before and after soaking in SBF for 14 and 28 days. As can be seen, white precipitates with an average particle size of 0.3 μm could be observed on the surface of akermanite powders with a flower morphology (Figure 9d–e). No precipitate was observed on the surface of akermanite samples before soaking in SBF solution (Figure 9a,b). The presence of phosphate and calcium peaks in the EDS spectra (Figure 9f) showed that these flower-like precipitates on the surface of akermanite samples are apatite (no phosphate peak was detected in Figure 9c). After increasing the soaking time up to 28 days, more and larger apatite particles (average size of 2–3 μm) covered the entire surface of the samples. Also, the results of EDS spectra confirmed the presence of apatite components in this specimen. The increase in the peak intensity of Ca and P can prove the formation of more apatite colony on the surface of akermanite.

**Figure 10:**
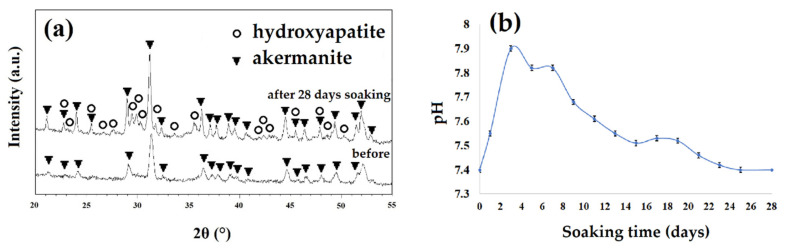
(**a**) The XRD patterns of the samples obtained after 20 h ball milling with subsequent annealing at 900 °C for 1 h before and after soaking in simulated body fluid (SBF) for 28 days and (**b**) the pH changes of samples as a function of time.

By considering the results of XRD patterns of samples before and after soaking in SBF solution (Figure 10a) and the pH changes (Figure 10b), it can be concluded that the white particles were hydroxyapatite. As seen, the maximum pH changes occurred in the second day of soaking the sample in SBF. During this soaking time, pH changed from 7.4 to 7.9 due to the breakdown of the Si-O-Si bonds and the release of ions from the ceramic structure to the SBF solution [31,32]. The release and replacement of the ceramic ions with H^+^ from the SBF solution can cause the formation of Si-OH groups and negative charges on the surface of specimen [32,33]. Consequently, Ca^2+^ ions with a positive charge can be absorbed on the surface of samples and decrease the pH in the solution (after 3 days of soaking). Subsequently, absorbing phosphate groups with a negative charge can occur and lead to the formation of an apatite layer on the surface of akermanite [31,32]. The release of Ca^2+^, Mg^2+^, and Si^4+^ ions from akermanite to the body environment, play an important role in cellular behavior [33,34]. Magnesium is an important mineral element in bone tissues and plays a key role in bone remodeling and skeletal development [31,32]. There is a growing interest in the concept of silicon’s essential role in bone formation and maintenance. Silicon (Si) improves bone matrix quality and facilitates bone mineralization [33,34]. Silicon supplementation in human body increases bone mineral density and improve bone strength [31,34]. On the other hand, Ca element causes cellular differentiation, proliferation, and extracellular matrix production [33,34]. As a result, a combination of Si, Mg, and Ca component in akermanite ceramic can be a promising candidate for bone tissue engineering applications.

The XRD patterns of the akermanite obtained after ball milling the initial materials for 20 h with subsequent annealing at 900 °C for 1 h before and after soaking in SBF for 28 days are illustrated in Figure 10a. The XRD patterns following 28 days soaking showed the characteristic peaks of apatite (JCPDS data file 01-076-0694) at 28.5, 29.2, and 31.5°. The results of pH changes are in a good agreement with the XRD, SEM, and EDS results.

**Figure 11:**
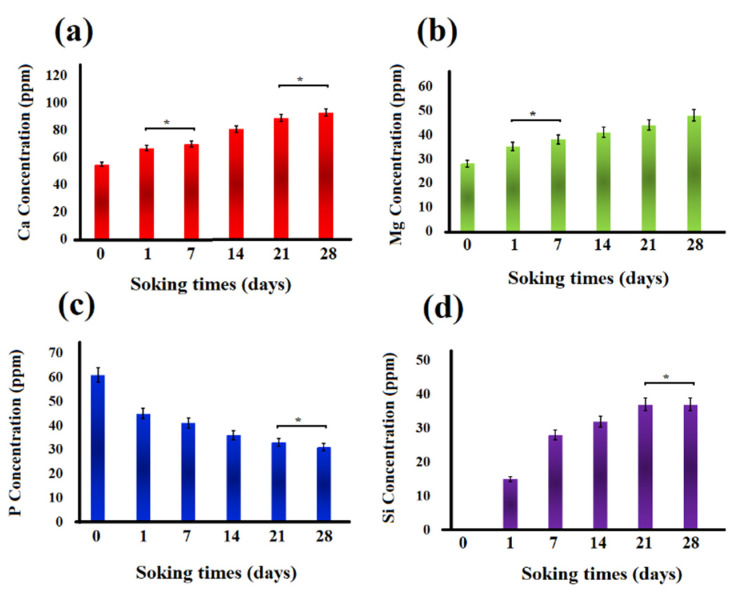
Ion concentration changes in the SBF solution: (**a**) Ca, (**b**) Mg, (**c**) P, and (**d**) Si ions. (* not significantly different).

Changes in the concentration of Ca, Mg, P, and Si ions in the SBF solution after soaking the akermanite ceramic for 0, 1, 7, 14, 21, and 28 days are shown in Figure 11. All the comparisons were performed between the samples which were significantly different. As seen, Mg, Ca, and Si ion concentration increased up to 28 days. This observation is in a good agreement with the pH changes and can be ascribed to the breakage of silicate bonds in akermanite structure which released Ca^2+^, Mg^2+^ and Si^4+^ ions into the solution. As can be seen in Figure 11, P ion concentration in SBF solution decreased up to 28 days as a result of the precipitation of Ca–P layer and apatite formation on the surface of akermanite ceramic which is in a good agreement with the results obtained from SEM and XRD analyses. It can be concluded that akermanite as one of the members of Ca–Mg-containing silicate bioceramics shows great potential as a biomaterial for bone repair and regeneration owing to its suitable bioactivity and mechanical properties.

## 4. Conclusions

This paper reports the synthesis of akermanite powder from talc, calcite and silica powders as the initial materials. Mechanical activation was used to synthesize pure nanocrystalline akermanite (Ca_2_MgSi_2_O_7_) powder. The optimum mechanical activation time and annealing temperature were 20 h and 900 °C for 1 h, respectively. The crystallite size of the akermanite powder was 35 nm calculated by the Williamson–Hall method. Akermanite tablets sintered at 1200 °C for 5 h showed a Young’s modulus of 3800 MPa, an ultimate compressive strength of 24.7 MPa, and a density of 2.489 g/cm^3^. Based on the XRD patterns, pH changes results, EDS spectrum, and SEM images of akermanite powders after a various soaking time in SBF, it was found that an apatite layer covered the entire surface of the akermanite powders. The results of the mechanical and physical properties as well as the in vitro behavior of the produced akermanite bioceramic exhibited a great potential of this ceramic to be used in bone tissue engineering applications.

## Supplementary Materials

The following are available online at https://www.mdpi.com/1996-1944/13/21/4887/s1, Figure S1: XRD pattern of silica powder, Figure S2: XRD patterns of ball milled samples at various time after annealing at 500 °C for 1 h, Figure S3: XRD patterns of ball milled samples at various time after annealing at 600 °C for 1 h, Figure S4: XRD patterns of ball milled samples at various time after annealing at 1000 °C for 1 h.
