# Supplementary material for: Fabrication, Mechanical Properties and In-Vitro Behavior of Akermanite Bioceramic
Source: Materials (Basel). 2020 Oct 30;13(21):4887. doi: 10.3390/ma13214887 (PMC7672555; doi:10.3390/ma13214887)
Supplement: Supplementary file 1 [file materials-13-04887-s001.zip › materials-971699-supplementary.docx]

*Supplementary Materials*

**Fabrication, Mechanical Properties and In-Vitro Behavior of Akermanite Bioceramic**

**Fariborz Tavangarian ^1,^*, Caleb A. Zolko ^1^, Sorour Sadeghzade ^1^, Marwan Fayed ^2^ and
Keivan Davami ^3^**

^1^ Mechanical Engineering Program, School of Science, Engineering and Technology, Pennsylvania State University, Harrisburg, Middletown, PA 17057, USA; [fut16@psu.edu](mailto:fut16@psu.edu); [caz5067@psu.edu](mailto:caz5067@psu.edu); [sxs2640@psu.edu](mailto:sxs2640@psu.edu)

^2^ Department of Mechanical and Nuclear Engineering, Pennsylvania State University, University Park, PA 16802, USA; [mqf5462@psu.edu](mailto:mqf5462@psu.edu)

^3^ Department of Mechanical Engineering, University of Alabama, Tuscaloosa, AL 35487, USA; [kdavami@eng.ua.edu](mailto:kdavami@eng.ua.edu)

***** Correspondence: fut16@psu.edu; Tel.: +1-717-948-6125

Received: date; Accepted: date; Published: date


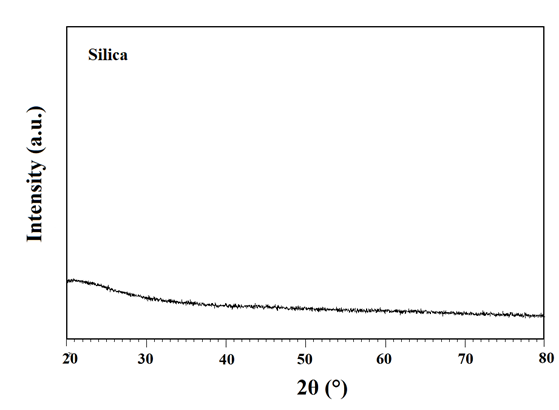


**Figure S1.** XRD pattern of silica powder.


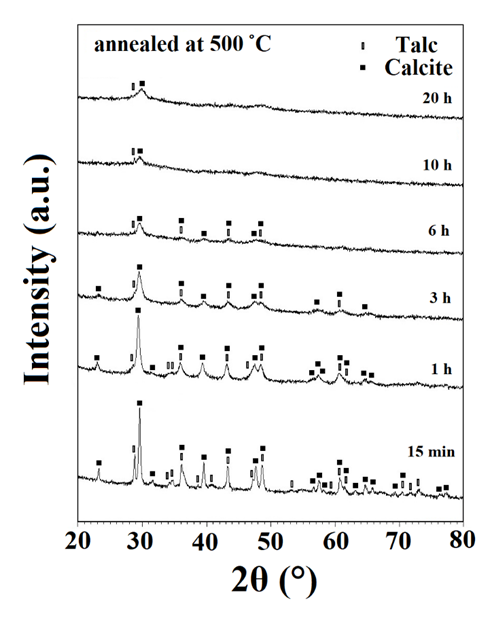


**Figure S2.** XRD patterns of ball milled samples at various time after annealing at 500 °C for 1h.


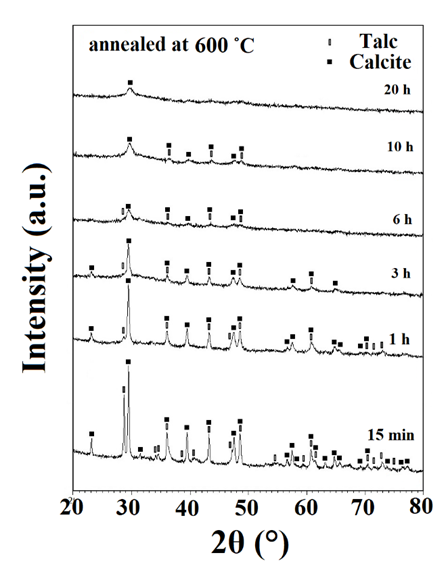


**Figure S3.** XRD patterns of ball milled samples at various time after annealing at 600 °C for 1h.


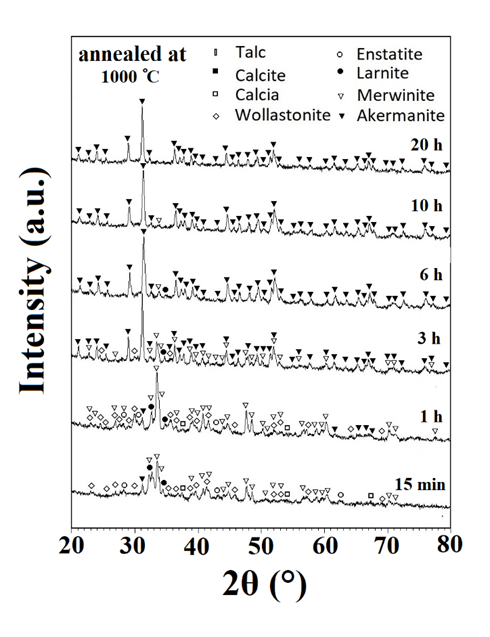


**Figure S4.** XRD patterns of ball milled samples at various time after annealing at 1000 °C.
